# Temporal phenotyping of circulating microparticles after trauma: a prospective cohort study

**DOI:** 10.1186/s13049-018-0499-9

**Published:** 2018-04-27

**Authors:** Matthias Fröhlich, Nadine Schäfer, Michael Caspers, Julia K. Böhm, Ewa K. Stürmer, Bertil Bouillon, Marc Maegele

**Affiliations:** 1 The Institute for Research in Operative Medicine, Faculty of Health, Department of Medicine, Witten/Herdecke University, Ostmerheimer Str. 200, D-51109 Cologne, Germany; 2 Department of Traumatology, Orthopaedic Surgery and Sports Traumatology, Cologne-Merheim Medical Centre (CMMC), Witten/Herdecke University, Campus Cologne-Merheim, Ostmerheimer Str. 200, D-51109 Cologne, Germany; 3 Department of Translational Wound Research, Centre for Biomedical Education and Research (ZBAF), Witten/Herdecke University, Stockumer Street 10, D-58453 Witten, Germany

**Keywords:** Microparticle, EDMP, PDMP, Trauma, Coagulation

## Abstract

**Background:**

After severe polytrauma the dynamic process of coagulation may deteriorate towards a trauma-induced coagulopathy (TIC) promoting a dramatic increase in morbidity and mortality. Recent evidence suggests that microparticles (MPs) play a pivotal role at the interface between cellular and plasmatic coagulation systems. However, the impact of MPs on functional coagulation has not been clarified yet in the setting of traumatic injuries. We assessed the temporal patterns of circulating MP concentrations including their cellular origin in the context of clinical presentation and global coagulation assays.

**Methods:**

Blood samples from 22 consecutive polytrauma patients (ISS ≥16) from 2015 were collected at hospital admission, after 24 and 72 h and compared to those from healthy individuals and minor injured patients with isolated extremity fractures. Flow cytometry (BD Accuri C6; Heidelberg/Germany) was used to determine MP concentrations and cellular origin using cell-specific markers (platelet derived (PDMP): CD42b^+^, CD61^+^, CD62p^+^; endothelial cell derived (EDMP): CD144^+^, CD62e^+^, CD144^+^/62e^+^). Results were correlated with clinical data and results from viscoelastic testing (ROTEM).

**Results:**

Twenty two polytrauma patients (17 males, age_median_ 60 yrs) with a median ISS 26.5 (IQR 14.5) were assessed. PDMP and EDMP concentrations increased significantly in polytrauma patients as compared to healthy individuals and minor injured patients. MP concentrations correlated with injury severity (CD144^+^: ρ_sp_ = 0.79, *p* < 0.001; CD42b^+^: ρ_sp_ = 0.61, *p* < 0.001). EDMP displayed a negative correlation with aPTT (CD144/62e^+^, ρ_sp_ = − 0.55, *p* < 0.05), INR (CD144/62e^+^, ρ_sp_ = − 0.61, *p* < 0.05) and ROTEM-INTEM CT (CD144/62e^+^, ρ_sp_ = − 0.68, *p* < 0.05) reflecting increased dynamics of clot formation and an overall procoagulative effect. Additionally, EDMP showed a negative association with FIBTEM values (10 min amplitude, maximum clot firmness) indicating a fibrinolytic potential.

**Discussion:**

In a small cohort, analysing most severly injured patients, the association of increased MP levels and altered coagulation parameters could be demonstrated. However, these findings are based on correlation analysis, which do not enable causel evidence. Therefore, further in-vitro studies are needed analysing the underlying pathomechanisms.

**Conclusion:**

In conclusion, this study could demonstrate that PDMP and EDMP levels increase significantly following polytrauma correlating with injury severity. Although severe coagulopathy was not observed, EDMP levels were associated with improved coagulation parameters suggesting their essential role for regulating blood coagulation after trauma.

## Background

The dynamic process of coagulation after traumatic injuries may deteriorate towards a trauma-induced coagulopathy (TIC) in which the early platelet dysfunction has a pivotal role [1]. Besides platelets, small cell-derived subcellular vesicles are suggested mediators of coagulation playing a crucial role at the conjunction of the cellular and plasmatic coagulation, circulating in both, healthy and diseased individuals [2, 3]. Although platelet-derived MP (PDMP) are the most abundant of all circulating MP, endothelial- and monocytes-derived MP (EDMP, MDMP) also participate in cellular signalling, coagulation and haemostasis by inducing platelet aggregation or developing a platelet- and fibrin-rich thrombus [3–6]. In detail, the procoagulative effects of MPs are mediated inter alia by externalized phospholipids such as phosphatidylserine (PS, while not all MP are PS-positive), tissue factor or von-Willebrand factor (vWF) [6, 7]. The role of MP as major coagulation stimulant became evident as ex-vivo generated PDMP were estimated having 50- to 100-fold higher procoagulant activity as activated platelets [8].

With respect to traumatic injuries, the comparability of studies on MP measurements remains challenging due to heterogeneity of trauma severity and injury pattern, the patient’s coagulation status, and the occurrence of TIC. For this reason, high level of both, procoagulant PS-positive PDMP and red-cell derived MP (RDMP) were reported by Curry et al., while Windelov and colleagues determined lower level of PS-PDMP in trauma patients who were characterized by impaired coagulation [9, 10]. Likewise, Matijevic and colleagues reported that the traumatic injury and the occurrence of TIC differentially affect MP quantity. Patients with TIC have significantly lower counts of MP than patients without. This observation was accompanied by a shift in the PDMP:EDMP ratio from healthy individuals (100:1) and trauma patients (6:1) and thus, increased EDMP, RDMP and leukocyte-derived MP (LDMP) as well as PDMP level tending to decrease after trauma when compared to healthy controls [11]. However, Balvers et al. measured lower PDMP and comparable EDMP and RDMP level after trauma compared to healthy individuals [12].

In this study, we determine MP quantity after polytrauma capturing a size range between 0.3 to 0.9 μm by a highly sensitive flow cytometry assay. The present study aimed to determine how (i) MP levels differ in polytraumatic patients from those in minor injured and healthy individuals, (ii) MP distribution changes over time (up to 72 h post-injury) and (iii) MP levels correlate with the coagulation systems displayed by functional coagulation assays.

## Methods

### Recruitment of patients and healthy volunteers

The present study is embedded in part in the multicentre Activation of Coagulation and Inflammation in Trauma II (ACIT II) study that obtained ethical approval from the Witten/Herdecke University (37/2005, 33/2015). Trauma patients admitted to the emergency department of the Cologne-Merheim Medical Centre were recruited that met the following criteria:

- adult (age ≥ 18 years),
- injury severity score (ISS) ≥16,
- less than two hours between injury and hospital admission,
- less than 2000 mls of pre-hospital fluid administration,
- no hospital transfer,
- exclusion of patients with severe liver disease, bleeding abnormalities and/or anticoagulant medication (excluding aspirin).

As reference group, minor injured patients (ISS < 16) had to meet the above-mentioned criteria with the exception of injury severity. Besides, healthy volunteers were included for blood donation if none of the subsequent exclusion criteria was appropriate:

- chronic disorders, anamnesis of thrombosis,
- viral, bacterial or fungal infections (within the last 14 days),
- blood, plasma or granulocyte donation (within the last 14 days),
- surgeries or dental treatments (within the last 14 days).

### Blood sampling and processing

Since blood coagulation is a dynamic process, which cannot be mirrored only by a single blood sampling at hospital admission, blood was furthermore drawn 24 and 72 h after arrival at the emergency department. For baseline values healthy volunteers donated blood once. Any kind of mechanical irritation or changes in temperature to the blood samples were avoided according to the recommendations of the International Society of Thrombosis and Haemostasis SSC Collaborative workshop [13]. Irrespective of the study group, EDTA blood was processed within 30 min according to a previously published protocol [9, 14]. Whole blood was centrifuged at 2500 x g for 10 min. In second stage, the supernatant was centrifuged at 13.000 x g for two minutes. The resulting platelet free plasma (PFP) was immediately frozen and stored at − 80 °C until further analysis.

### Blood count, coagulation parameters and rotational thrombelastometry (ROTEM®)

Blood count and standard coagulation parameters such as activated partial thromboplastin time (aPTT) and international normalised ratio (INR) were obtained from the ACL TOP system (Instrumentation Laboratory Company, Bedford, USA). Rotational thrombelastometry (ROTEM®, TEM International GmbH, Munich, Germany) was used within 30 min following blood sampling at 37 °C according to manufacturer’s recommendations. EXTEM, INTEM and FIBTEM (TEM international GmbH, Munich, Germany) assays were used. EXTEM and FIBTEM evaluate the extrinsic activation using recombinant tissue thromboplastin. FIBTEM thereby analyses the fibrinogen-mediated component of clot strength blocking the activation of platelets by Cytochalasin D. INTEM depicts the contact based intrinsic pathway. The following variables were recorded: clotting time (CT), clot formation time (CFT), a-angle, amplitude after 5 (A5) and 10 min (A10) and maximal clot firmness (MCF).

### Flow cytometry

Flow cytometry with cell-specific markers was used to determine the MP quantity and their cellular origin in the blood of polytrauma patients, minor injured and healthy individuals. MP were defined as particles measuring 0.3 to 0.9 μm. Prior to sample analysis, the threshold and upper limit of MP size was standardised using forward scatter (FSC) and sideward scatter (SSC) characteristics of latex beads (Megamix Plus, BioCytex, Marseille, France) according to manufacturer’s recommendation. This enabled to exclude the influence of small platelets and to restrict the MP size. The lower detection limit was achieved using a combination of SSC and fluorescence based thresholds.

PFP was thawed on melting ice. Cell-specific antibodies for thrombocytes or endothelial cells were added to 30 μl plasma and incubated 20 min at room temperature in the dark. For the determination of platelet origin, we used fluorescein isothiocyanate (FITC)-labelled anti-CD42b and peridinin chlorophyll (PerCP) Cy5.5-labelled anti-CD61. For MP derived from activated platelets, phyocerythrin (PE)-labelled anti-CD62p was used. For the determination of endothelial origin, we used FITC-labelled anti-CD144 and for MP derived from activated endothelial cell PE-labelled anti-CD62e. Additionally, allophycocyanin (APC)-labelled Annexin V was used as marker of procoagulant phosphatidylserine (PS) in all measurements. All antibodies were obtained from BD Bioscience (Becton Dickinson, Heidelberg, Germany). Following incubation, 500 μl 0.2 μm-filtrated PBS-diluted Annexin binding buffer (BD, Heidelberg, Germany) were added and samples were analysed immediately on Accuri C6 (BD, Heidelberg, Germany) for one minute. Results are presented as count of positive stained events within the MP gate per microliter of PFP.

### Statistics

Results are presented as median and interquartile range (IQR). Nonparametric Kruskal-Wallis test was performed to compare MP level between healthy volunteers and injured patients. Linear regression was performed to evaluate the influence of injury severity on MP level. Repeated measurements over time (for patients sustaining a polytrauma) were analysed using the Friedman test. Furthermore, MP level were correlated with clinical data and with results from thrombelastometric measurements using Spearman’s rank correlation analysis.

A *p* < 0.05 was considered statistically significant. Statistical analyses were performed using SPSS statistics version 21 (IBM Corp., Armonk, NY) and GraphPad Prism version 7.00 for Windows (GraphPad Software, La Jolla California USA).

## Results

### Patients and healthy individuals

Overall, 22 polytrauma patients admitted to the emergency department met the inclusion criteria. Almost all patients sustained a blunt trauma causing injuries with a median severity of ISS = 26.5. Almost 60% of the included trauma patients had severe injuries (ISS ≥25). Overall, mainly affected were the thoracic region (72.7%), followed by the head/neck (63.6) and extremities (40.9%, Table 1). More than 1/3 of all patients (*n* = 8) showed shock indications at hospital admission with a shock index > 1.0. Furthermore, a subset of 10 patients received blood products (any type) and thereof four were massively transfused (more than 10 units RBCs within 24 h). At day 28 following the traumatic injury the mortality rate accounted for less than 20%.

**Table 1:** Characteristics of patient and healthy individual collective

	Polytrauma patients (*n* = 22)	Monotrauma patients (*n* = 10)	Probands (*n* = 10)
Age, median (IQR) (years)	60 (31.5)	57.5 (32)	45.5 (25.5)
Male sex, n (%)	17 (77.3)	4 (40)	6 (60)
Blunt trauma, n (%)	21 (95.5)	10 (100)	–
ISS, median (IQR)	26.5 (14.5)	4 ***	0 ***
AIS_Abdomen_ ≥ 3, n (%)	4 (18.2)	0	0
AIS_Thorax_ ≥ 3, n (%)	16 (72.7)	0	0
AIS_Head/neck_ ≥ 3, n (%)	14 (63.6)	0	0
AIS_Extremity_ ≥ 3, n (%)	9 (40.9)	2 (20)	0
Vital signs at admission			
Heart rate, median (IQR) (bpm)	100 (44.5)	–	–
Systolic blood pressure, median (IQR) (mmHg)	122 (38.8)	–	–
Patient with shock index > 1.0, n (%)	8 (36.4)		
Blood parameters at admission			
Haemoglobin, median (IQR) (g/dl)	12 (2.2)	14 (1)	–
Platelet count, median (IQR) (/nl)	197 (49)	249 (97)	–
INR, median (IQR)	1.12 (0.1)	1.03 (0.1)	–
INR > 1.2, n (%)	4 (18)	–	
aPTT, median (IQR) (s)	30 (9.1)	24.5 (3.2)	
Fibrinogen, median (IQR) (g/l)	1.8 (0.6)	–	–
Base excess, median (mmol/l)	−1.7	–	–
ROTEM measures (study day 1)			
EXTEM CT, median (IQR) (s)	68 (19)	65.5 (5.5)	–
EXTEM CFT, median (IQR) (s)	90 (35)	75 (14)	–
EXTEM MCF, median (IQR) (mm)	61 (7)	65.5 (3)^*^	–
FIBTEM CT, median (IQR) (s)	60 (15)	60.5 (5.8)	–
FIBTEM A10, median (IQR) (s)	11.5 (4)	14.5 (4.3)^*^	–
FIBTEM MCF, median (IQR) (mm)	12 (5)	17.5 (7.3)	–
Transfusion requirement (first 24 h)^a^			
RBC, median (IQR) (unit)	7 (14.5)*	0	–
FFP, median (IQR) (unit)	5 (9.3)*	0	–
Platelet, median (IQR) (unit)	0.5 (1.8)	0	–
Mortality (28 days), n (%)	4 (18.2)**	0	–

^a^Only patients considered who received any type of blood products (10 patients received RBC, thereof 6 patients receiving FFP and 5 receiving platelets, respectively). Statistical significances were marked with asterisks (*p < 0.05, **p < 0.01, ***p < 0.001)

Ten patients with isolated extremity fracture (median ISS of four) and ten healthy volunteers were recruited representing minor injured and healthy controls, respectively. Both groups approximately corresponded in age compared to the group of polytrauma patients.

### Functional and conventional coagulation assays

ROTEM measures were in the normal reference range for the entire polytrauma cohort (Table 1). However, these patients had significant lower extrinsic maximum clot firmness and FIBTEM amplitudes 10 min after clot initiation than patients with monotrauma. With respect to conventional coagulation analyses, aPTT and INR were slightly prolonged in polytrauma patients without reaching statistical significance (Table 1). Coagulopathic tendencies, defined as INR > 1.2 at hospital admission, was observed in 18% (*n* = 4) of all 22 trauma patients.

### MP quantities in healthy individuals and traumatic patients

**Fig. 1:**
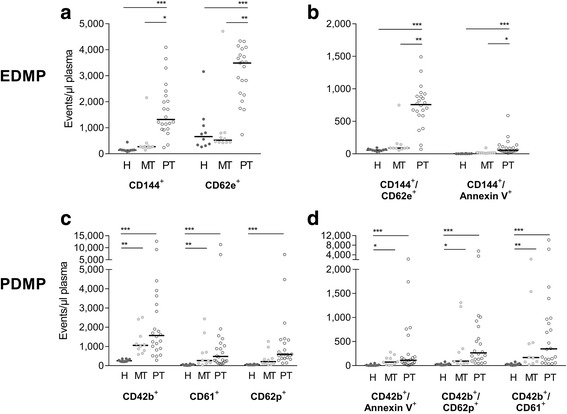
MP quantities derived from platelets (PDMD) end endothelial cells (EDMP) are depicted for healthy individuals (H) and patients sustaining a monotrauma (MT) or polytrauma (PT). **a**,**b**) CD144 - VE-Cadherin, CD62e - e-Selectin; **c**, **d**) CD42b -  GP1b, CD61 - GP3a, CD62p - p-Selectin; *p < 0.05, **p < 0.01, ***p < 0.001

**Fig. 2:**
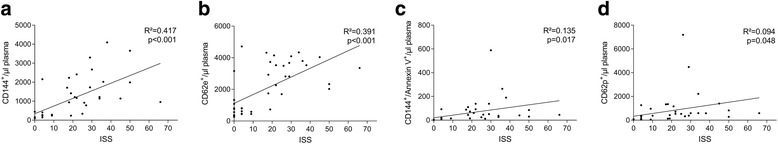
Linear regression of MP quantities per microliter at hospital admission and injury severity; **a**, **c**) CD144 - VE-Cadherin; **b**) CD62e - e-Selectin; **d**) CD62p - p-Selectin

Compared with healthy individuals, the polytraumatic injury led to an increase of circulating MP. Mainly 0.3–0.9 μm MP with endothelial (CD144^+^) and activated endothelial origin (CD62e^+^) corresponded with injury severity. While healthy individuals and patients with isolated extremity fractures had approximately comparable low EDMP quantities (median values for CD144^+^ and CD62e^+^ in healthy vs. monotrauma (141.5 vs. 273 particles/μl plasma and 660.5 vs. 522.5 particles/μl plasma, Fig. 1a)), patients following a polytrauma (median values: CD144^+^ = 1318.5 and CD62e^+^ = 3489 particles/μl plasma, Fig. 1a) showed significant higher EDMP level. Similar differences were measured for double-positive labelled CD144^+^/CD62e^+^ particles, which were 8.5 and 13.1fold increased (based on the median) in polytrauma than in monotrauma patients and healthy individuals, respectively (Fig. 1b). The observation of increasing MP quantities between healthy individuals and mono−/polytrauma was confirmed by a positive linear regression explaining about 40% of the variation of EDMP by the injury severity (ISS) (Fig. 2a-b).

Platelet-derived MP (CD42b^+^ and CD61^+^ events) gradually increased being low in healthy individuals and ascending to monotrauma and further to polytrauma (Fig. 1c). Particles derived from activated platelets (CD42b^+^/CD62p^+^) were determined as being 10- and 3.5-times higher in the groups of severe injured and light casualty than in healthy probands (based on median; healthy vs. monotrauma vs. polytrauma: 26 vs. 93.5 vs. 265 particles/μl plasma, Fig. 1d). However, injury severity explained at maximum 10% of CD62p^+^ MP as being the only significant linear regression result for PDMP (R^2^ = 0.09, *p* < 0.048, Fig. 2d).

### Time-dependent MP characteristics in polytrauma

**Fig. 3:**
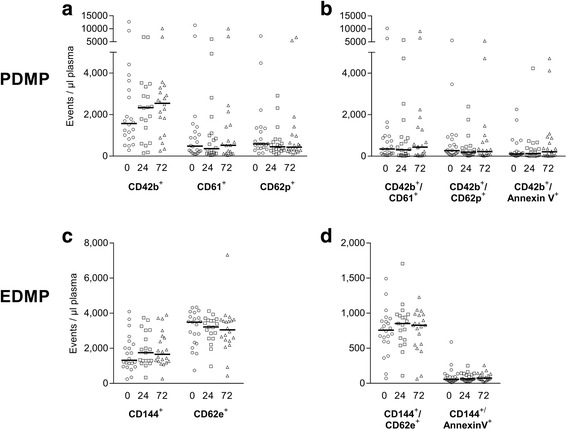
Time-dependent MP development of polytrauma patients from hospital admission, 24 and 72 h afterwards (marked as circles, quadrats and triangle symbols, respectively). **a**,**b**) CD42b - GP1b, CD61 - GP3a, CD62p - p-Selectin; **c**, **d**) CD144 - VE-Cadherin, CD62e - e-Selectin. No statistical significance was observed

Within 72 h after trauma the total number of EDMP and PDMP did not change significantly in polytraumatised patients (Fig. 3). Considering single patients, as well increasing as decreasing MP numbers could be observed, thus each patient displayed a unique pattern after injury. These differing courses occurred without any measurable influence of transfused blood products or surgical interventions.

### Correlations between MP, injury severity, coagulation parameters and mortality

The numbers of EDMP and PDMP correlated well with the injury severity displayed by the ISS (CD144^+^: ρ_sp_0 = 0.79; CD42b^+^: ρ_sp_ = 0.61, *p* < 0.001) as shown in Table 2. EDMP presenting procoagulant PS on their surface correlated negatively with the aPTT (ρ_sp_ = − 0.55) and INR (ρ_sp_ = − 0.61) indicating an accelerating effect on the intrinsic and extrinsic coagulation pathways. Correspondingly, EDMP presenting any endothelial marker were negatively associated with the INTEM CT, while the extrinsic pathway was not affected. Furthermore, negative associations with the plasmatic coagulation displayed FIBTEM could be observed. The number of PDMP did not correlate with the platelet count or any conventional coagulation tests. PDMP from activated platelets (CD42^+^/CD62p^+^) were associated with increased D-dimer levels. PDMP numbers linked to INTEM CT, although only correlation of CD42b^+^ MP reached statistical significance. In addition, the EDMP (CD62p^+)^ and activated PDMP (CD42b^+^/AnnexinV^+^ and CD42^+^/CD62p^+^) correlated positively with 28 days mortality.

**Table 2:** Correlations between circulating MP count and injury severity, laboratory coagulation parameters and functional coagulation testing

		EDMP				PDMP					
		CD144^+^	CD62e^+^	CD144^+^/ CD62e^+^	CD144^+^/ Annexin V^+^	CD42b^+^	CD61^+^	CD62p^+^	CD42b^+^/ Annexin V^+^	CD42b^+^/ CD62p^+^	CD42b^+^/ CD61^+^
Injury	ISS	0.793***	0.653***	0.776***	0.763***	0.611***	0.635***	0.740***	0.603***	0.657***	0.605***
	AIS_Head/Neck_	0.500**	0.573**	0.632***	0.512**	0.389*	0.435**	0.580***	0.402**	0.501**	0.425**
	AIS_Thorax_	0.685***	0.589***	0.616***	0.613***	0.374*	0.405**	0.533***	0.421**	0.435**	0.392*
	AIS_Abdomen_	0.433***	0.303	0.234	0.376*	0.209	0.146	0.155	0.161	0.159	0.140
Outcome	Mortality 28 days	0.04	0.279	0.501*	−0.170	0	0.421	0.501*	0.481*	0.461*	0.421
Standard Coagulation	Platelets	− 0.185	− 0.002	− 0.073	0.073	− 0.075	− 0.045	− 0.084	− 0.175	− 0.066	− 0.073
	aPTT	−0.183	− 0.316	− 0.393	−0.554*	0.062	0.273	0.273	0.299	0.322	0.259
	INR	−0.090	−0.131	− 0.405	−0.607*	0.020	0.064	0.047	0.034	0.099	−0.019
	D-dimer	−0.181	0.107	−0.214	−0.427	− 0.125	0.463*	0.387	0.407	0.467*	0.441*
Functional Coagulation (ROTEM)	EXT CT	0.053	0.169	−0.078	−0.077	− 0.188	−0.193	− 0.097	−0.187	− 0.070	−0.189
	EXT MCF	−0.400*	−0.256	− 0.278	−0.203	0.030	0.185	0.038	0.137	0.151	0.233
	INT CT	−0.547**	−0.396*	− 0.476**	−0.681**	− 0.453*	−0.243	− 0.357	−0.148	− 0.269	−0.179
	INT MCF	−0.310	−0.250	− 0.232	−0.026	0.080	0.181	0.029	0.093	0.133	0.240
	FIB A10	−0.551**	−0.414*	− 0.396*	−0.284	− 0.129	−0.015	− 0.169	−0.077	− 0.050	−0.058
	FIB MCF	−0.459*	−0.384*	− 0.346	−0.271	0.099	0.202	0.078	0.109	0.177	0.266

Values are shown as Spearman correlation coefficient (*ρ*) between the variables. Statistical significances were marked with asterisks (*p < 0.05, **p < 0.01, ***p < 0.001)

## Discussion

This observational study could demonstrate that

(i) MP levels increased significantly following polytrauma compared to minor injured patients and healthy individuals.
(ii) within 72 h after injury, the MP distribution did not change significantly depending on the time.
(iii) EDMP correlated negatively with coagulation parameters indicating an accelerating effect on the intrinsic and extrinsic coagulation pathways.
(iv) mainly EDMP correlated with injury severity.

MPs, which are released from various cell types, have been described in both, diseased and healthy individuals. The MP majority (> 80%) derive from platelets, while smaller quantities derive from endothelial cells and leukocytes [2]. In the analysed healthy individuals, this relation of PDMP to EDMP could be reproduced. Variations in MP’s concentration or cellular origin have been reported for various medical conditions [11, 15]. However, recent literature is split over the role of MP following major trauma. As well increased unspecified MP and PDMP levels were reported as others determined decreased values following trauma [9–12]. More consistently was reported, that patients with impaired coagulation or TIC show lower levels of PS-PDMP [10, 11]. Interestingly, while Matijevic et al. observed an increase of EDMP levels independently of the presence of TIC, Park et al. found EDMP only in one of 59 injured patients [11, 16]. In comparison to the presented data, previous studies included patients with a wide range and in mean lower injury severity [9, 10, 12]. Focusing on most severely injured patients (median ISS 26.5) and comparing them to minor injuries and healthy individuals, we could demonstrate that PDMP and EDMP levels increase depending on and correlating to the injury severity. Particularly, positive correlations between the ISS and endothelial MP determined by CD144 and CD62e could be shown by the present study indicating that these markers might serve as promising biomarker for injuries of the epithelium and injury severity. Similarly, CD144^+^ vesicles had been suggested as potential marker for endothelial injuries in other clinical settings [17].

In healthy individuals only low levels of circulating EDMP were detectable. Following polytrauma, we observed significant 8.5 and 13.1-fold EDMP increased compared to monotrauma patients and healthy individuals, which is in line with the PROMMTT cohort in which 20-fold higher EDMP numbers were measured [11]. This endothelial reaction to trauma appears double-edged on their influence on the clot formation. On the one hand, as shown the present study, EDMP correlated negatively with the INTEM clotting time and PS^+^-EDMP even correlated negatively with aPTT and INR (Table 2). In addition to TF^+^- and PS^+^-bearing PDMP, which are known to have a higher procoagulant activity than platelets [18], our results show the EDMP’s procoagulant potential. Previously, it has been described that an increasing concentration of EDMP shortens the plasma clotting time in a presumable TF-/FVII-dependant manner [6, 19]. Jy and colleagues could demonstrate that a subset of EDMP carry unusually large vWf multimers, promoting platelet aggregation and increasing the stability of the aggregates formed [5]. On the other hand, PS-negative EDMP correlated negatively with FIBTEM A10 and FIBTEM MCF, displaying a certain anticoagulant effect. Lacroix et al. described that in pathological settings EDMP bear tissue plasminogen activator and therefore postulated EDMP as “a circulating source for fibrinolysis” [20]. In addition, subsets of EDMP have membrane-bound endothelial Protein C receptors [21]. Consequently, activated membrane-bound Protein C retains its anticoagulant activity [21], which is in line with findings by Matijevic and colleagues, as higher EDMP levels correlated negatively with thrombin generation [11]. However, we could not observe this association in the presented study as an impaired thrombin generation could be expected to affect EXTEM values. Nevertheless, MP and especially EDMP express both, activators and inhibitors of coagulation, and therefore seem to stabilise the haemostatic balance [22].

As recommended in current guidelines, preventing the development and treating TIC is of outmost importance for patients’ outcome following severe trauma [23]. Since increased MP levels seem to improve haemostasis and might represent an endogenous compensation mechanism, the additional administration of pro-haemostatic agents might lead to spontaneous clotting resulting in thromboembolic events. Aside from trauma, elevated levels of circulating MP are associated with thrombotic events in cancer patients [24, 25]. However, in the present cohort we observed only one thromboembolic event 14 days after trauma. As patients were treated according to current guidelines and guided by viscoelastic testing, the risk of adverse events due to increased MP levels seems manageable. In contrast, MP levels might be helpful determining the point when thromboprophylaxis is indicated. Furthermore, the administration of artificial MP might become an additional tool in haemostatic therapy. Nevertheless, this remains fully speculative and further studies have to clarify MPs’ signalling and detailed function.

However, several PDMP populations correlated positively with 28 days mortality, which is in contrast to previous studies [9–11]. In a cohort analysed by Curry et al., mortality correlated negatively with PS-positive PDMP values [9]. Going into detail of the fatal cases in the presented study, two patients died early after trauma showing low MP values, while one patient, having the highest overall MP values, died 14 days after trauma by pulmonary embolism. Therefore, it appears obvious, that the presented cohort might be too small to draw any further conclusions regarding patient outcome. Interestingly, besides the mentioned studies suggesting a protective effect of MP due to effects on the coagulation system [9–11], a recent study could demonstrate reduced MP levels in patients with ARDS (Acute Respiratory Distress Syndrome) [26].

As could be expected, PDMP and EDMP levels were increased at hospital admission of trauma patients. Comparable to a previous study, we could not observe any statistically significant trend of MP levels over 72 h after trauma [27]. Although our trauma cohort was more concise regarding injury severity compared to the patients analysed by Park et al., still differences in injury pattern and in the early in-hospital treatment might contribute to this MP development [27].

Several limitations have to be admitted interpreting the presented study. The results derive from a small cohort, but in comparison to previous studies focusing on the relevance of MP following trauma, we were able to include comparably serious injured patients. Certainly, a larger cohort might have led to more significant results regarding the PDMPs procoagulatoric potential. Furthermore, the presented conclusions are based on correlations, which do not provide causal evidence. Although general trends in the posttraumatic increase of MPs are comparable to previous studies, absolute numbers differ in part distinctively [7, 9–12, 27]. This underlies the importance to strive for a standardisation in pre-analytical variables and flow-cytometric protocols according to the recommendations by the International Society on Thrombosis and Haemostasis [13].

## Conclusion

In conclusion, this study could demonstrate that PDMP and EDMP levels increase significantly following polytrauma correlating with injury severity. Although severe coagulopathy was not observed, EDMP levels were associated with improved coagulation parameters suggesting their essential for regulating blood coagulation after trauma. However, further studies analysing the procoagulative and fibrinolytic potential are needed.

## Abbreviations

aPTT: Activated partial thromboplastin time
ARDS: Acute respiratory distress syndrome
CFT: Clot formation time
CT: Clotting time
EDMP: Endothelial derived microparticle
FSC: Forward scatter
IQR: Interquartile range
ISS: Injury severity score
LDMP: Leukocyte derived microparticle
MCF: Maximum clot firmness
MDMP: Monocyte derived microparticle
MP: Microparticle
PDMP: Platelet derived microparticle
PFP: Platelet free plasma
PS: Phosphatidylserine
RBCs: Red blood cells
RDMP: Red-cell derived microparticle
ROTEM®: Rotational thrombelastometry
SSC: Sideward scatter
TF: Tissue factor
TIC: Traumatic induced coagulopathy
vWF: von Willebrand Factor
